# Sample-to-answer lateral flow assay with integrated plasma separation and NT-proBNP detection

**DOI:** 10.1007/s00216-024-05271-3

**Published:** 2024-04-09

**Authors:** Dan Strohmaier-Nguyen, Carina Horn, Antje J. Baeumner

**Affiliations:** 1 Institute of Analytical Chemistry, Chemo- and Biosensors, University of Regensburg, 93053 Regensburg, Germany; 2 Roche Diagnostics GmbH, 68305 Mannheim, Germany

**Keywords:** Lateral flow assay, Blood plasma separation, Sedimentation, Point-of-care diagnostics, Sample-to-answer

## Abstract

Through enabling whole blood detection in point-of-care testing (POCT), sedimentation-based plasma separation promises to enhance the functionality and extend the application range of lateral flow assays (LFAs). To streamline the entire process from the introduction of the blood sample to the generation of quantitative immune-fluorescence results, we combined a simple plasma separation technique, an immunoreaction, and a micropump-driven external suction control system in a polymer channel-based LFA. Our primary objective was to eliminate the reliance on sample-absorbing separation membranes, the use of active separation forces commonly found in POCT, and ultimately allowing finger prick testing. Combining the principle of agglutination of red blood cells with an on-device sedimentation-based separation, our device allows for the efficient and fast separation of plasma from a 25-µL blood volume within a mere 10 min and overcomes limitations such as clogging, analyte adsorption, and blood pre-dilution. To simplify this process, we stored the agglutination agent in a dried state on the test and incorporated a filter trench to initiate sedimentation-based separation. The separated plasma was then moved to the integrated mixing area, initiating the immunoreaction by rehydration of probe-specific fluorophore-conjugated antibodies. The biotinylated immune complex was subsequently trapped in the streptavidin-rich detection zone and quantitatively analyzed using a fluorescence microscope. Normalized to the centrifugation-based separation, our device demonstrated high separation efficiency of 96% and a yield of 7.23 µL (= 72%). Furthermore, we elaborate on its user-friendly nature and demonstrate its proof-of-concept through an all-dried ready-to-go NT-proBNP lateral flow immunoassay with clinical blood samples.

**Graphical Abstract:**

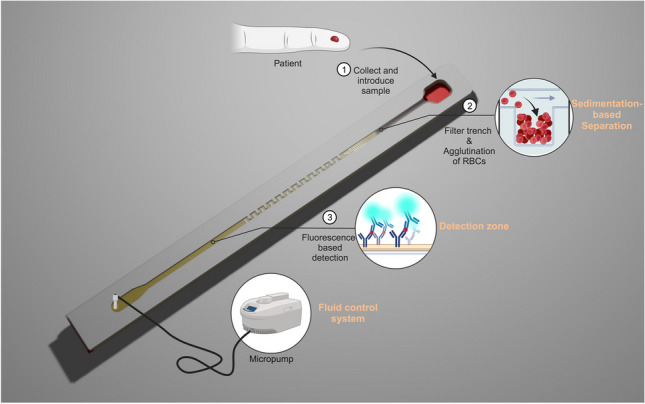

**Supplementary Information:**

The online version contains supplementary material available at 10.1007/s00216-024-05271-3.

## Introduction

Medical professionals commonly employ blood tests as a primary diagnostic method to detect various conditions. This procedure entails examining a small blood sample for any deviations or abnormalities in the individual’s biochemical profile, which may indicate the presence of a pathological disorder [1]. Whole blood is one of the most complex and relevant body fluids that is used in clinical analysis [2]. Its analysis typically requires costly bench-top instruments and skilled personnel, making it unsuitable in resource-limited surroundings [3]. Moreover, conventional blood tests are relatively complex and time cumbersome (> 1 h) requiring large sample volumes [4, 5]. The utilization of POCT technology has exhibited the ability to scale down the size of laboratory instruments and assays, resulting in economically favored, reproducible, and faster detection systems. The success of POCT systems lies in the integrated separation mechanism in order to circumvent bulky bench-top centrifuges to generate human plasma, removing the necessity for sample handling, transport, and storage. The plasma extraction through a patented separation membrane such as asymmetric polysulfone membrane (APM), Fusion 5, and others is the gold standard. Its well-defined pore size retains the blood cells in the membrane while the plasma can be further analyzed. Especially, in low-setting surroundings, the separation membrane has been shown advantageous. Previous studies have indicated that the accuracy of testing results heavily relies on the quality of plasma, which can be negatively impacted by blood cell–related issues like hemolysis and leukolysis [6, 7]. The separation of blood cells is primarily aimed at achieving a reproducible detection read-out as the presence of red blood cells and their lysed fragments can obstruct the visibility of test and control lines, leading to inaccurate results [8]. Nonetheless, employing the separation membrane also brings about limitations, including blood clogging [9], potential interactions with analytes [10], as well as relatively high sample volumes, caused by the membrane’s high dead volume. This, in turn, might cause discomfort for the patient, as the blood needs to be drawn from a vein rather than through a finger prick. Therefore, recent studies have concentrated on plasma separation techniques suitable for small finger prick sample volumes [11–13], which can generally be categorized into active and passive methods [14]. Active separation includes external fluid control forces, whereas, in passive separation, no external forces are required for the separation process, making the system more suitable for the POCT environment [15]. The majority of the reported separation techniques, however, come with three major disadvantages. Firstly, expensive and cumbersome bench-top instruments are required, hindering the application in low-resource settings [16]. Secondly, the device involves sophisticated geometric structures [17], posing a challenge in achieving consistent fabrication and hindering the scalability process [18]. Thirdly, these techniques exhibit limited efficiency in separating plasma from high hematocrit samples, necessitating off-chip dilution of whole blood before analysis, thereby introducing cost and complexity to the procedure and diluting the target analyte, which presents a challenge in precisely detecting such minimal levels of analytes, especially when working with small finger prick volumes [19–21]. Affordable technologies capable of achieving efficient blood-plasma separation at the point of need, while ensuring reliability and effectiveness, are in demand. Plasma separation by sedimentation has been used for decades and relies on the gravitational density disparities between plasma and blood cells (ρ_RBCs_ = 1100 kg/m^3^, ρ_WBCS_ = 1050–1090 kg/m^3^, and ρ_plasma_ = 1030 kg/m^3^) [14]. Blood cells in whole blood tend to sediment based on their differing densities, allowing the plasma to remain on top as a result of this separation process [22]. Sedimentation offers advantages as it eliminates the need for expensive equipment and highly trained personnel. One major limitation of this process, however, is the time it takes for blood cells to settle towards the bottom. Scaling down the dimension and integrating the process into a microfluidic system shortens the sedimentation time as the blood cells are effectively separated within shorter distances. Dimov et al., for example, observed that the gravitational force acting on blood cells is considerably greater than that on plasma within a filter trench, which allows the plasma and blood cells to segregate into an upper and a lower layer [23]. However, the self-contained system exhibited an unstable separation rate, and it necessitated relatively low flow rates to achieve 100% filtration efficiency. Such low flow rates are impractical when dealing with larger blood volumes. Yang et al. further developed the integration of the gravitational separation mechanism in a microfluidic design [24]. The device requires a heater to create a vacuum or a low-pressure environment for fluid transportation, but this introduces considerable variability and can be further affected by temperature fluctuations from the external environment. Additionally, only 2 µL of plasma could be separated from 10 µL whole blood. Therefore, we proceeded to explore the filter trench while considering the impact of an aggregation agent that facilitates the clustering of red blood cells during the sedimentation phase, thereby enhancing the sedimentation rate. Consequently, we designed a lateral flow channel assay including a sedimentation-based erythrocyte separator that separates red blood cells (RBCs) from plasma in only 10 min. A filter trench not only acts as a physical barrier for the blood cells but also enables the RBCs to sediment, leading to high separation efficiency with low-cost ingredients. By improving the filter trench depth and sedimentation time, the separation efficiency can be increased. Eventually, the assessment of the practicality of utilizing our device for the separation of clinical blood samples in the context of an immunochemical test was conducted. By controlling the fluid movement via a micropump, the separated plasma passes through the trench and is analyzed in a fluorescence sandwich-based immunoreaction.

## Materials and methods

The biotinylated capture antibody (polyclonal NT-proBNP sheep-IgG-biotin, cAb), antigen (NT-proBNP (1–76) amid) in whole blood, probe antibody (monoclonal NT-proBNP mouse-IgG), probe antibody–modified fluorescence nanoparticles (Ab-fluorescence NPs), albumin (97%), and poly streptavidin (pSA) were provided by Roche Diagnostics GmbH (Mannheim, Germany). Hydrochloric acid (HCl, 0.1 M, 1 M), sodium chloride (NaCl, p.a.), bovine serum albumin (BSA, > 96%), poly(diallyldimethylammonium chloride) (PDDA, M_w_ 200,000–350,000, 20 wt.% in H_2_O), poly(acrylic acid, sodium salt) solution (average M_w_ 15,000, 35 wt.% in H_2_O), ethylenediaminetetraacetic acid (EDTA, ≥ 98.5%), sodium hydroxide (NaOH, 1 M), poly(ethylene glycol)-block-poly(propylene glycol)-block-poly(ethylene glycol) (Synperonic® PE/P84), sodium azide, and Tween 20 (> 97%) were supplied from Sigma-Aldrich (www.sigmaaldrich.com). HetaSep™ (www.stemcell.com) was utilized as agglutination agent.

### Lateral flow channel fabrication with filter trench

The lateral flow channel was composed of an inlet, a channel, a filter trench, a detection zone, and an outlet that was connected to the micropump (Fig. S1). For the construction of the lateral flow channel, a slightly modified procedure of Yang et al. was used [24]. The platform is built-up out of four layers: (1) the substrate (Melinex®329, 175 µm), (2) a capillary-given spacer (Melinex®329, 250 µm), both were purchased from Dupont Teijin Films (www.dupontteijinfilms.com), (3) the filter trench-given foam spacer was customized by ATP Adhesive Systems (atp-ag.com), and (4) the cover foil Hostaphan RN 100 was purchased from Mitsubishi Polyester Film (www.m-petfilm.de). The capillary-given spacer and the filter trench-given spacer were coated with double-sided adhesive tape on both sides, which was supplied by Henkel-Adhesives (www.henkel-adhesives.com). The channel (dimension: 90 mm × 1.5 mm × 0.28 mm), filter trench (dimension: 10 mm × 1.5 mm × 3 mm), the inlet (dimension: 4.5 mm × 3 mm × 0.28 mm), and outlet (dimension: Ø 1.5 mm) were designed by using the CorelDraw 2016 software and were then engraved.

To create the all-in-one, single-step immunoassay, the bio-recognition line on the substrate (Melinex®329, 175 µm) was patterned perpendicular to the flow direction with 1-mm-wide lines of a polyelectrolyte-poly streptavidin multilayer using the layer-by-layer approach before assembling the device. Briefly, poly streptavidin (500 µL, 10 mg∙mL^−1^) and polydiallyldimethylammonium chloride (500 µL, 0.5% (w/v)) were mixed at pH 7.4 and 150 mM NaCl to produce PDDA-poly streptavidin complexes. To assemble the detection zone, the PDDA-poly streptavidin complexes and polyacrylic acid (0.5% (w/v), pH 4.55, PAA) were alternately deposited for 60 s onto the plastic slide through a shape-giving mold. The repetition of this alternative coating procedure was carried out for four cycles to create the detection zone. Capture Abs at a concentration of 2.5 µg∙mL^−1^ and the Ab-fluorescence NPs at a concentration of 2% (w/v) were individually dispensed onto the immunoassay support, with 2 µL of each reagent. For the plasma separation, 6 µL of the aggregation agent was dispensed in the sample application zone. The dispensed reagents were then dried using a drying cabinet at a temperature of 50 °C for a duration of 10 min. Following the drying process, the support, spacer, and cover foil were assembled as shown in Figure S1 of the Electronic Supplementary Material (ESM).

### Human blood sample

Samples of human blood from healthy donors were provided from Roche Diagnostics (Mannheim, Germany) in vacutainers with 7.2 mg K2 ethylenediaminetetraacetic acid (EDTA).

### Quantification of the plasma purity

For the quantification of the plasma purity, we followed the procedure of Sneha Maria et al. with slight changes [25]. Briefly, the plasma purity was examined in the lateral flow channel by comparing the grayscale intensities of the plasma obtained by the proposed device (Ig_trench_) and the plasma obtained from the centrifuge (Ig_centrifuge_). As RBCs are darker in color than plasma, their presence in the sample can reduce the grayscale intensity of the channel. Hence, the plasma purity was expressed as followed:

1 $$purit{y}_{plasma}=100\%* \frac{{I}_{g\_trench}}{{I}_{g\_centrifuge}}$$

### Quantification of the recovered plasma volume

The volumes of the plasma present in the channel and the volume of the plasma in whole blood after centrifuge were measured, by comparing the distance of transported plasma in the channel, and the following formula was applied:

2 $$volum{e}_{plasma}={h}_{channel}*{w}_{channel}*{d}_{plasma}$$

where, h_channel_, w_channel_, and d_plasma_ represent the channel height, channel width, and traveled distance of plasma, respectively.

### Performance of the bioassay

A sandwich immunoassay with spiked blood samples was carried out to investigate the assay performance of the lateral flow channel assay. The samples were prepared in human whole blood through dilution of an AG stock solution (0–9000 pg∙mL^−1^) for analysis. For the immunoassay, 25 µL of the spiked sample was applied on the sample application area. The sample was immediately transported to the filter trench with a flow rate of 60 µL∙min^−1^ by the external vent control. After the separation time of 10 min, the separated plasma was further transported to the outlet with a flow rate of 2 µL∙min^−1^, crossing the detection zone. The immunoassay was performed at room temperature and fluorescence signals could be obtained after 35 min. For the calculation of the limit of detection (LOD), the logistic fit parameter for the lower curve asymptote A and the standard deviation of the blank SD (blank):

3 $$LOD = A + 3.3 * SD(blank)$$

The concentration of the antigen in the sample is determined by correlating the fluorescence intensity to a calibration curve constructed using known antigen concentration standards.

### Supporting instruments

The drying processes were carried out using a drying cabinet set at a temperature of 50 °C (FED 400 E2, www.binder-world.com). The fluid control equipment and software used in this study were custom-made and supplied by Roche Diagnostics GmbH (Mannheim, Germany) (Fig. S2a). The fluorescence images were captured using a fluorescence microscope equipped with a LINOS lens (www.excelitas.com) and an HTC camera featuring a Sony CCD sensor ICX285AL (www.sony.com) (Fig. S2b). Illumination was provided by a XENON XBO R 100W/45 OFR lamp (www.osram.com), and specific excitation and detection filters (633-nm excitation and 685-nm detection) were utilized (www.semrock.com). The imaging software used for data acquisition was provided by Roche Diagnostics (Mannheim, Germany). The fluorescence images were taken with an exposure time of 25 ms. Image processing and data analysis were carried out with ImageJ and Origin 2021.

## Results and discussion

### Design and operational principle behind the lateral flow channel assay device

**Fig. 1:**
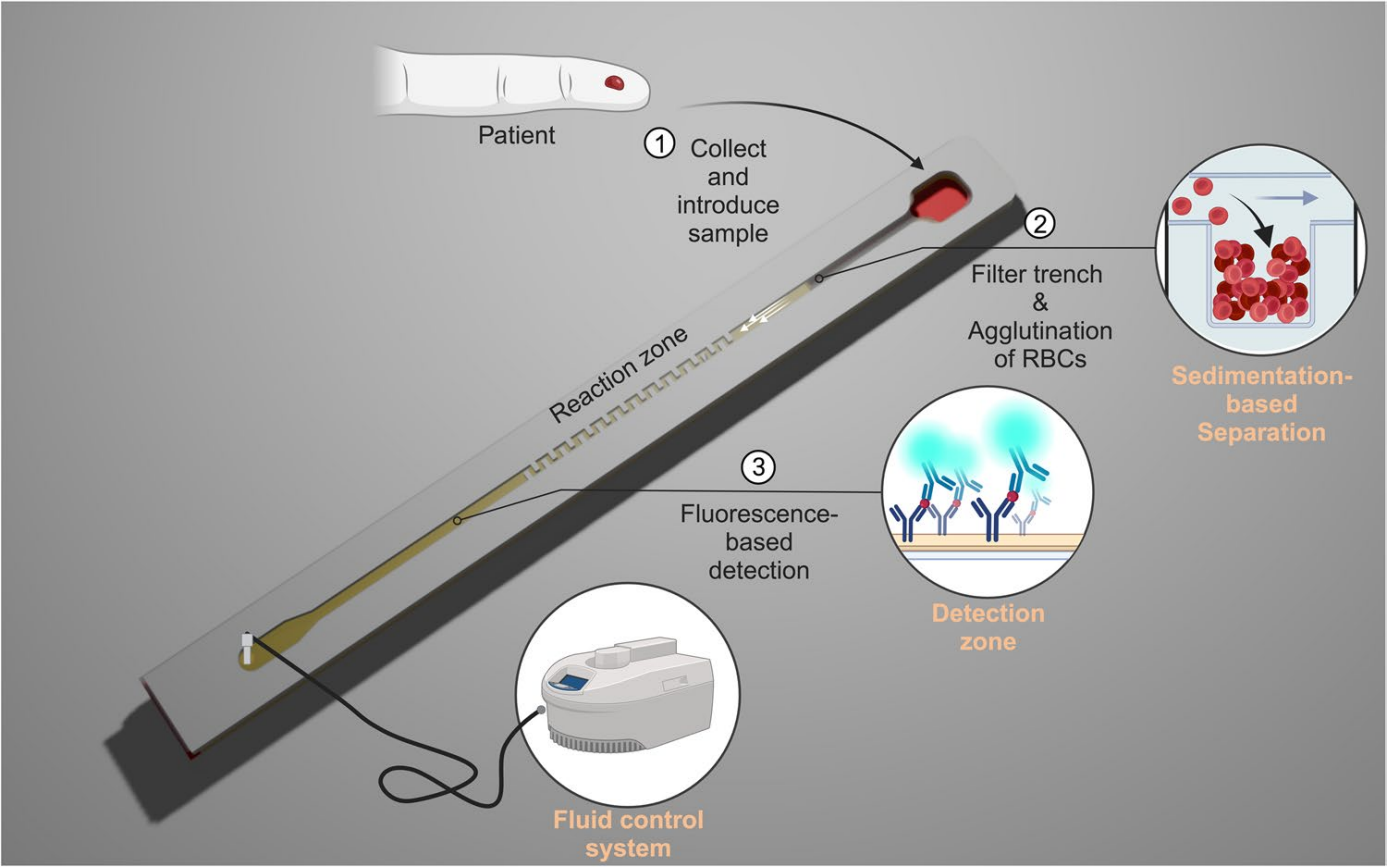
Schematic (not to scale) of the lateral flow channel assay with integrated plasma separation. After introducing the sample to the inlet (1), the sample is transported to the filter trench by the fluid control system. In the filter trench, the RBCs are aggregated and separated from the plasma through sedimentation (2). The separated plasma is then moved from the plasma separation module towards the reaction zone, reconstituting the probe antibodies and initiating the immunoreaction. In the detection zone, streptavidin is immobilized on the bottom of the channel for capturing the biotinylated sandwich complex (3). Adapted from “PDMS microfluidic chip fabrication”, by BioRender.com (2023). Retrieved from https://app.biorender.com/biorender-templates

The integrated blood-plasma separation biosensing system should exhibit reproducible functionality, fast and high separation efficiency, and compatibility with finger prick sample volumes. Our setup enables sensitive analyte detection within ~ 20 min with minimal user intervention. The proposed device includes the sample application port, the filter trench, the straight capillary channel with a mixing zone, the detection zone, and the outlet valve that is connected to the micropump (Fig. 1). After applying the blood sample at the inlet, the micropump controls the fluid movement, guiding the sample towards the filter trench. Within the filter trench, the agglutination agent prompts the red blood cells to aggregate. Subsequently, the sample was halted for a specified duration to ensure stable sedimentation of the blood cells. Finally, the microprocessor selectively activated the pump to sequentially move the sample from the plasma separation module towards the reaction zone, reconstituting the probe antibodies and initiating the immunoreaction. In the detection zone, streptavidin is immobilized on the bottom of the channel for capturing the biotinylated sandwich complex. Through the process of blood cell separation, we aim to minimize the impairment of the fluorescence signal and reduce any potential obstructions caused by the presence of hemoglobin in red blood cells. In our device, the separated plasma is further transported to the outlet and then the detection zone is fluorescently analyzed for quantitative signals (Fig. S3).

### Plasma separation efficiency

**Fig. 2:**
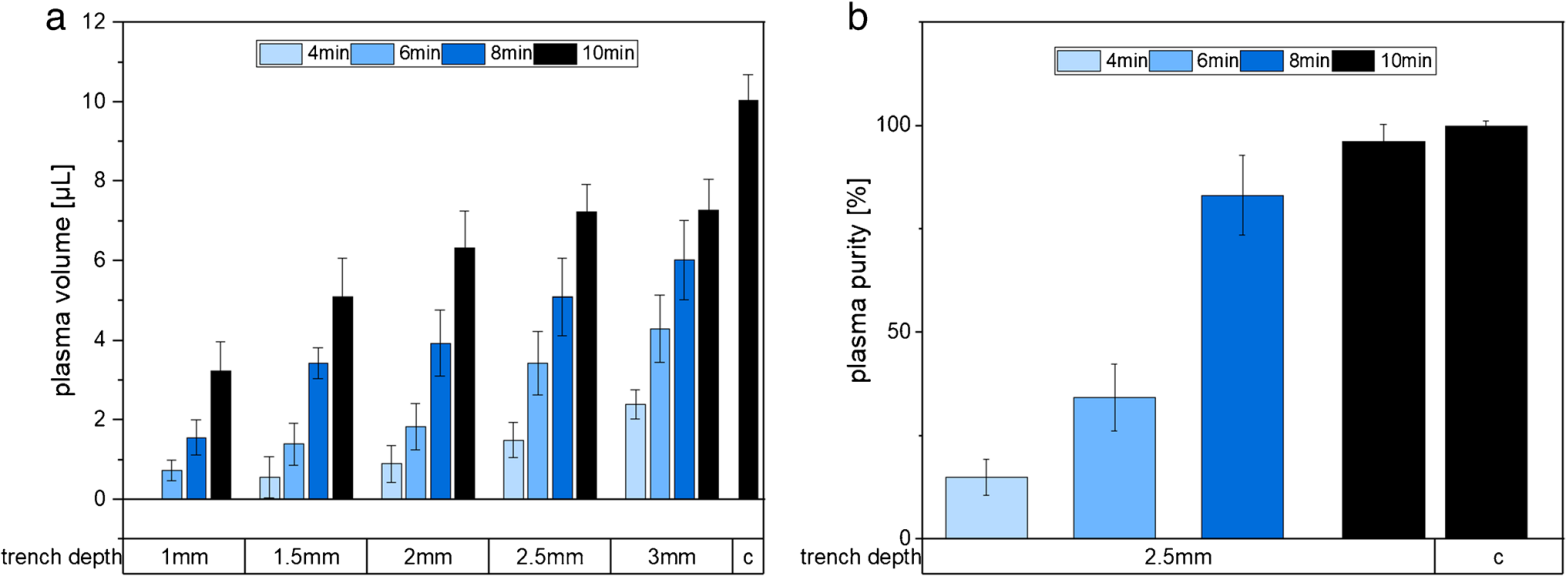
Optimization of the separated plasma volume (**a**) and optimization of the plasma purity (**b**) with respect to the trench depth and the time for separation. “c” represents the plasma separation through centrifugation. The lateral flow channel was loaded with 25 µL of whole blood (hematocrit, 44%), and the purity as well as the volume of plasma obtained was measured. Error bars represent the standard deviation (*n* = 4)

For the separation process to work efficiently, the time that blood cells remain in the trench must be long enough to cause sedimentation and entrapment within the trench. Therefore, the impact of various parameters including trench depth and separation time on the efficacy of plasma separation to enhance the effectiveness of the trench filter were studied (Fig. 2a). Specifically, a volume of 25 µL undiluted whole blood was introduced into the platform and subsequently transported to the filter trench for varying durations of separation. The experimental results confirmed that longer sedimentation times led to an increase in the volume of obtained plasma. This outcome was in line with expectations, as the extended sedimentation period allowed the blood cells more time to settle, resulting in a greater volume of separated plasma. Additionally, increasing the depth of the trench also led to an increase in plasma volume. In fact, increasing the depth of the filter trench was found to enhance the sedimentation effect, as gravity had a significant impact on blood cells in the trench [22]. Insufficient or no separation of blood was observed in cases of shorter sedimentation times (1–2 min) and shallow trenches (1–2 mm). For the sedimentation time of 10 min, the trench depth of 2.5 mm and 3 mm yielded the highest plasma volumes, measuring 7.23 µL and 7.29 µL, respectively. This corresponds to a plasma yield of 72% and 73% compared to that achieved through centrifugal separation (= 10.05 µL). This suggests that increasing the trench depth at this dimension does not lead to an enhancement in plasma yield. The overall observed decrease in relative plasma volume can be attributed to the presence of minor volumes of plasma that remain in the filter as dead volume, which cannot be effectively separated. This phenomenon accounts for the reduced overall plasma volume obtained in the process. Additionally, it was noted that a minor fraction of whole blood experienced non-specific adsorption at the device inlet, resulting in a decrease in the overall plasma volume obtained. This also highlights the discrepancy in plasma volume between the proposed separation method and the centrifugal separation process. Given the relatively comparable obtained plasma volumes for both trench depths, the choice for further experimentation was at 2.5 mm due to the potential reduction in material costs associated with this specific trench depth. All-in-all, these findings contribute to the understanding of key factors influencing plasma separation in the proposed system and offer guidance for optimizing the design and operation parameters of the lateral flow channel.

### Plasma purity

The evaluation of plasma purity was performed by analyzing the variation in grayscale intensity within a defined section of the channel containing the separated plasma [25]. This analysis aimed to assess the effectiveness of plasma purification in both the proposed approaches and the conventional centrifugation process. By comparing the grayscale intensities, we were able to quantify the level of impurities present in the obtained plasma and determine the efficiency of the separation methods. Due to the darker color of RBCs, the grayscale intensities in the presence of RBCs will be significantly lower compared to a sample without RBCs. The grayscale intensities of the separated plasma showed that the purity of the plasma obtained from the proposed approaches is similar to the plasma obtained using centrifugation (Fig. 2b). Furthermore, since photometric absorption was not applicable with the tested volume, visual inspection of the separated plasma samples showed no visible signs of hemolysis, such as discoloration or the presence of red blood cell remnants [26]. This provided additional evidence supporting the absence of hemolysis during the plasma separation process, further ensuring the integrity and quality of the obtained plasma samples. However, it should be noted that platelets, which have lower sedimentation rates due to its smaller size (diameter ~ 2 µm) [27], may be present in the extracted plasma. It is worth mentioning that the presence of platelets does not notably interfere with the fluorescence read-out, consistent with existing findings [22, 28]. Nonetheless, should there be a requirement to identify platelets within the sample, a range of imaging techniques can be employed [29]. Overall, the findings demonstrate the effectiveness of the proposed approach in achieving high-quality plasma separation [11, 12, 23] and shows its potential for various biomedical applications.

Table 1 provides a comprehensive summary and comparison of the performance characteristics of the proposed device for blood separation in contrast to the conventional centrifuge-based method, highlighting its potential for practical and efficient blood separation applications. Included are key parameters such as separation efficiency, plasma volume yield, processing time, and equipment requirements, which demonstrate that the proposed device achieved comparable performance. The device demonstrated high separation efficiency and yields a substantial volume of plasma with minimal loss. Additionally, the processing time was significantly reduced, offering a time-efficient alternative. Furthermore, the proposed device required minimal equipment, making it a cost-effective and accessible solution. However, it should be noted that the overall higher mean error in the separation efficiency and the separation yield may arise from variations in the manual fabrication of the test.

**Table 1:** Comparison of performance characteristics of the separation techniques

	Separation technique			
	Filter trench		Centrifuge	
Performance characteristics	Mean	sd	Mean	sd
Plasma purity	96%	6%	Set to 100%	2%
Plasma volume obtained	7.23 µL	0.74 µL	10.05 µL	0.63 µL
Separation yield	72%	7%	Set to 100%	6%
Time for extraction	10 min		15 min	
Required equipment	Portable pump		Bench-top centrifuge	

25 µL undiluted whole blood (hct 44%) was used for each test. Samples were run in triplicates and the mean and the standard deviation are reported. Results were normalized to the average volume of plasma and purity obtained from centrifugation (10.05 µL, 100%) (*n* = 3)

### Combination of on-device plasma separation with analyte detection

**Fig. 3:**
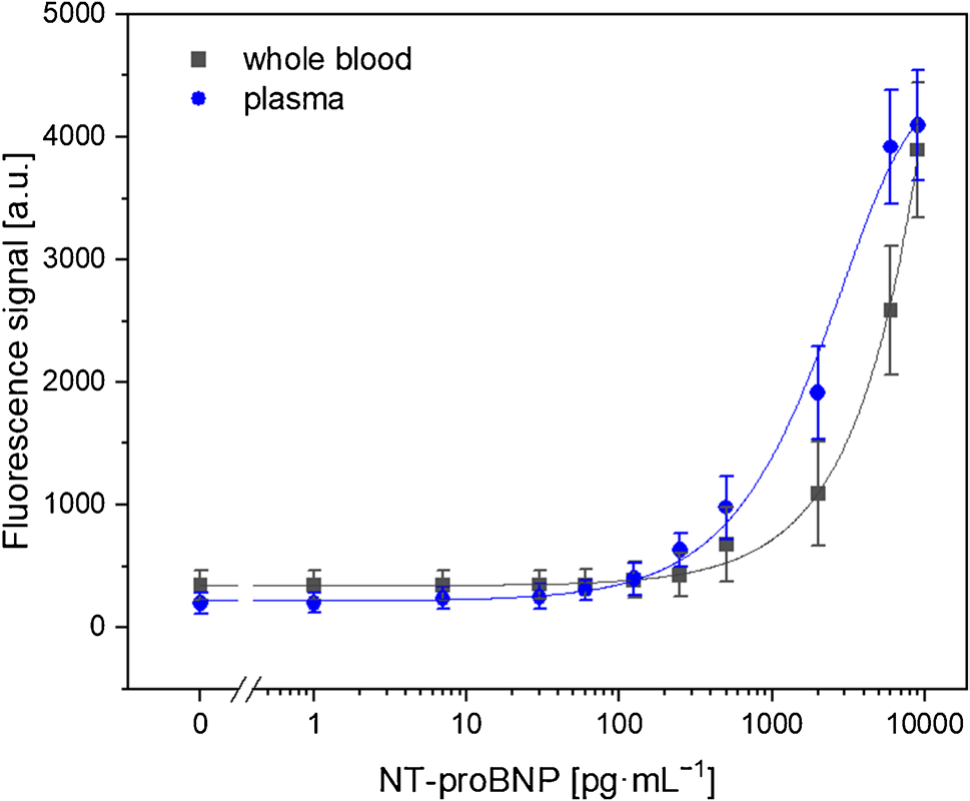
Plot of fluorescence intensity against logarithm of antigen concentration. Fluorescent read-out of the analyte in 25 µL whole blood with logistic fit (black line) and using the integrated plasma separation with logistic fit (blue line). Standard deviations were calculated based on four parallel measurements on four different LFAs, while outliers were removed after *Q*-test (confidence interval 95%). Error bars represent mean values ± 1σ (*n* ≥ 4)

Following the proof-of-principle and optimization of the blood separation, the proposed separation mechanism was implemented into a sandwich-based lateral flow assay. The platform was tested with real human blood samples, spiked with different concentrations of NT-proBNP (Fig. 3). The 25 µL undiluted whole blood is introduced to the sensor and guided towards the filter trench. Along the way, the dry-spotted biotinylated capture Abs are resolubilized, initiating the immunoreaction. After 10 min of sedimentation time in the filter trench, the separated plasma is further transported to the dry-spotted Ab-fluorescence NPs, initiating the formation of the sandwich complex. The sample is then transported to the detection site where the channel’s bottom is immobilized with streptavidin. Finally, the presence of the biotinylated sandwich complex can be detected at the detection zone, resulting in highly sensitive and specific detection of the target analyte. The immunoassay without blood separation and the proposed on-chip plasma separation showed similar curve shapes, suggesting comparable dynamic ranges. For the measurements with integrated blood separation, a limit of detection (LOD) of 365 pg∙mL^−1^ with a mean error of 18% was calculated in comparison to 283 pg∙mL^−1^ and 14% mean error for the centrifugation-based plasma preparation. At the sample application zone and in the filter trench, resulting from manual fabrication variations are likely to be the reason for the higher LOD for our platform.

But may also be caused by the lower plasma separation yield (Table 1). At the same time, the assay without blood separation demonstrated inferior signal responses in the analyte concentration range above 500 pg∙mL^−1^ and below 9000 pg∙mL^−1^. Moreover, the biosensor would provide a LOD of 1680 pg∙mL^−1^, and a mean error of 26%, hence, resulting in an about fivefold higher LOD for whole blood on-chip analysis. This discrepancy can be attributed to the adsorption of blood cells, particularly RBCs, onto the test line, leading to the absorption of the fluorescence signal due to their broad absorption spectra [30]. In addition, higher background signals were observed at low analyte concentrations (< 500 pg∙mL^−1^), resulting in the data points to not be statistically significantly different with the sample size of *n* = 4 used. Still, the achieved LOD value and the statistically significant data fall within the threshold range used to evaluate heart failure severity and the risk of hospitalization [31]. The improved detection limit was expected due to the prevention of blood cell adsorption in the detection zone and the interaction with the read-out. Thus, the on-strip blood separation drastically improves the detection capabilities. Currently, commercially available NT-proBNP tests, such as the Cobas h 232 provided by Roche Diagnostics, demonstrate superior temporal and analytical performance with a LOD of 60 pg∙mL^−1^ and a detection time of only 12 min. This suggests that there is still potential for enhancement in our developed platform. Nonetheless, the assay showcased in this study presents a notable benefit by demanding a notably smaller blood volume (25 µL compared to 150 µL). This reduction is attributed to the diminished dead volume stemming from the unused filtration membrane and nitrocellulose membrane. This not only increases convenience for the patient but also allows for sample collection via a simple finger prick, eliminating the necessity for skilled personnel and significantly cutting down costs for testing. As a result, our platform is well suited for home-testing and point-of-care applications in low-resource settings.

## Conclusion

In summary, we proposed a self-contained lateral flow channel assay with an integrated sedimentation-based separation of red blood cells from plasma, enabling sensitive quantification of the HF biomarker NT-proBNP in undiluted whole blood. The platform relies on the gravity-based sedimentation of agglutinated RBCs in a filter trench allowing for the subsequent immunoreaction to take place without blood cell interference and also demonstrates resilience against potential clogging that can arise in pore-based filtration mechanisms. The proposed biosensor is capable not only of handling small quantities of undiluted whole blood (25 µL), which improves the patients’ comfort as the sample volume can be conveniently obtained via a finger prick, but also possesses a cost-effective production approach since no complex geometric µm-structures are required. This facilitates consistent manufacturing and seamless scalability of production. Utilizing minimal hardware, including a portable pump and a fluorescence detection camera, the platform demonstrated the desired control over the flow rate and compatibility with a fluorescence immunoassay, while retaining the straightforward one-step detection feature found in conventional LFAs. Consequently, this uncomplicated plasma separation method can easily be applied to the POCT in clinical and home testing settings. Future enhancements will focus on further reducing sample volume requirements by avoiding analyte loss in both the inlet and the filter trench through an optimization of the test design’s geometric structure. Furthermore, moving towards real-world application, an evaluation of the separation efficiency with varying hematocrit levels in blood samples will be essential. In the end, the system’s applicability extends beyond NT-proBNP detection and can be applied to a broader range of clinically relevant blood biomarkers.

### Supplementary Information

Below is the link to the electronic supplementary material.

Supplementary file1 (DOCX 1546 KB)
