# Supplementary material for: Sample-to-answer lateral flow assay with integrated plasma separation and NT-proBNP detection
Source: Anal Bioanal Chem. 2024 Apr 9;416(13):3107–15. doi: 10.1007/s00216-024-05271-3 (PMC11068687; doi:10.1007/s00216-024-05271-3)
Supplement: Supplementary file 1 — Supplementary file1 (DOCX 1546 KB) [file 216_2024_5271_MOESM1_ESM.docx]

Supporting Information for

Sample-to-answer lateral flow assay with integrated plasma separation and NT-proBNP detection

Dan Strohmaier-Nguyen^a^, Carina Horn^b^, Antje J. Baeumner^a^*

^a^ University of Regensburg, Institute of Analytical Chemistry, Chemo- and Biosensors, 93043 Regensburg

^b^ Roche Diagnostics, 68305 Mannheim, Germany

**Table of contents**

- Text S1. Material and Methods
- Figure S1-S3

**Text S1. Material and Methods**

**Fluid control system**

The fluid control consisted of a metal housing that houses 1) a LFA mold, 2) an Arduino Due board, 3) and a miniaturized vacuum pump. The fluid control system was linked to a computer via USB and managed using an Arduino code. To initiate the immunoassay fhe fluid control was mounted on top of the outlet port of the LFA. The setup consists of a micropump that is mechanically positioned on the test strip using a suction cup. The pump works on the basis of the piezo effect. There are two piezoceramics in a pump that deform when a voltage is applied, allowing the transportation of liquid. These piezo actuators enable the pumping of liquid in a specific direction.

**Supporting figures**

| 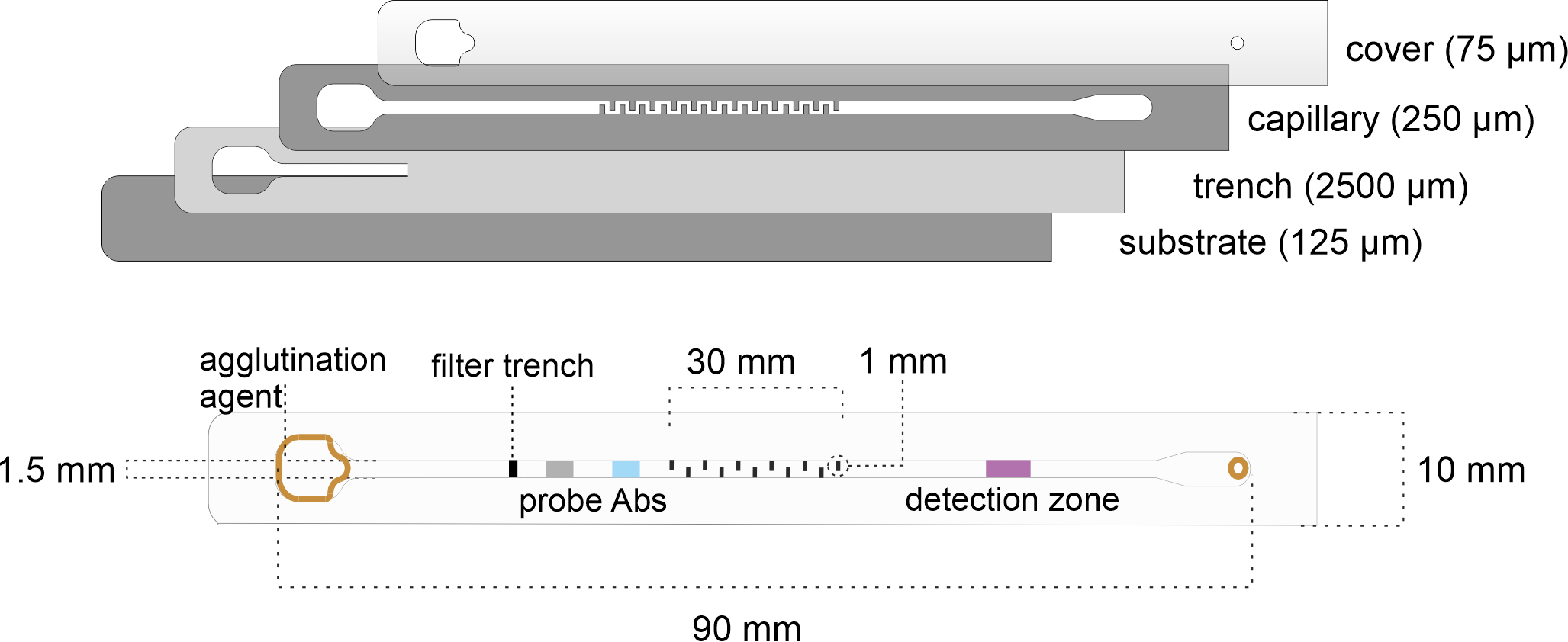 |
| --- |

**Fig. S1**: Lateral flow build-up. The lateral flow channel consists of four layers: (1) the substrate, (2) the trench, (3) the capillary and (4) the cover. Purple shape represents the detection zone, brown shape represents the agglutination agent, gray and blue shapes represent probe capture and probe detection antibody labeled fluorescence NP.

| **a**  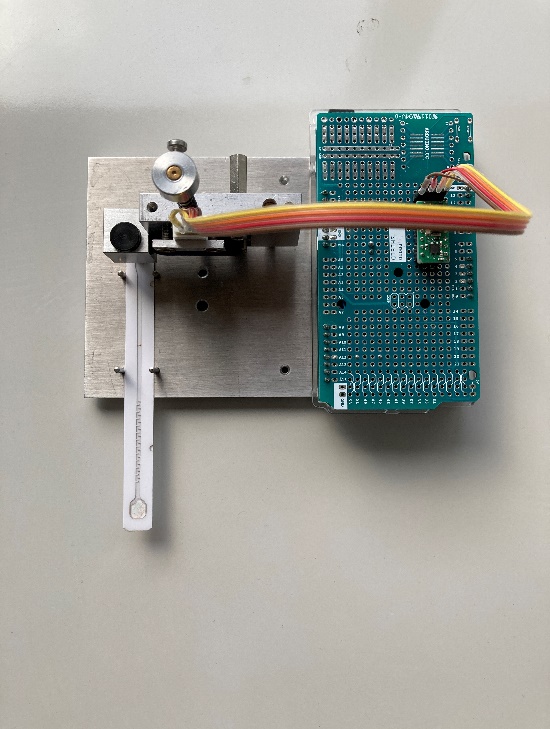 | **b**  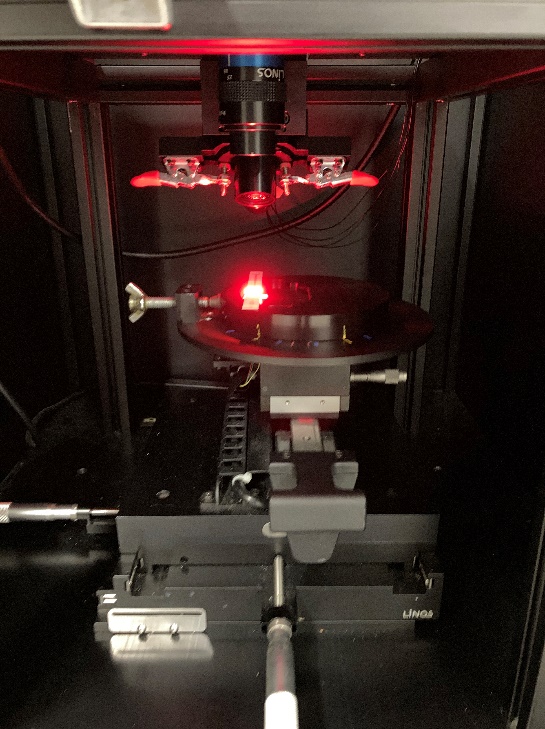 |
| --- | --- |

**Fig. S2**: Experimental set-up. Photograph of the micropump (a) and the fluorescence microscopy (b).

| 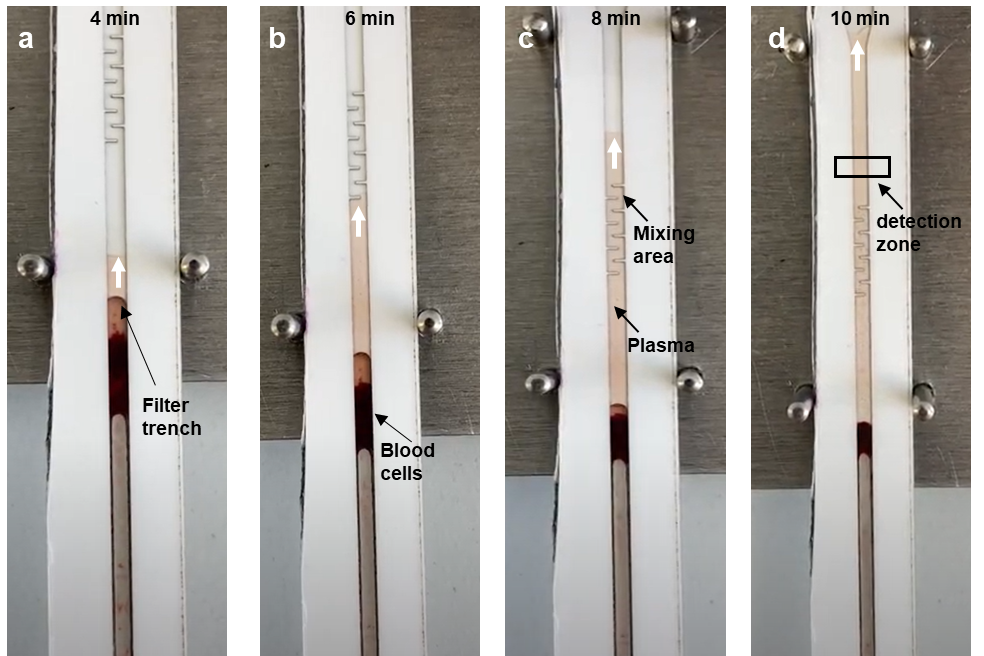 |
| --- |

**Fig. S3**: Snapshots showing plasma extraction along the plasma separation channel within 10 minutes. Agglutinated red blood cells are trapped inside the filter trench, eventually resulting in separation of plasma (a). The separated plasma is routed to the mixing area (b). Mixing of plasma and rehydrated probe-specific fluorescent-labeled antibodies, result in the formation of immune-complexes (c). The biotinylated immune-complex is captured in the streptavidin-rich detection zone for the fluorescence read-out (d).
